# Clinical implementation of MRI-based organs-at-risk auto-segmentation with convolutional networks for prostate radiotherapy

**DOI:** 10.1186/s13014-020-01528-0

**Published:** 2020-05-11

**Authors:** Mark H. F. Savenije, Matteo Maspero, Gonda G. Sikkes, Jochem R. N. van der Voort van Zyp, Alexis N. T. J. Kotte, Gijsbert H. Bol, Cornelis A. T. van den Berg

**Affiliations:** 1 Department of Radiotherapy, Division of Imaging & Oncology, University Medical Center Utrecht, Heidelberglaan 100, Utrecht, 3508 GA The Netherlands; 2 Computational Imaging Group for MR diagnostics & therapy, Center for Image Sciences, University Medical Center Utrecht, Heidelberglaan 100, Utrecht, 3508 GA The Netherlands

**Keywords:** Prostate cancer, Radiotherapy, Magnetic resonance imaging, MR-only treatment planning, Delineation, Contouring, Segmentation, Artificial intelligence, Deep learning

## Abstract

**Background:**

Structure delineation is a necessary, yet time-consuming manual procedure in radiotherapy. Recently, convolutional neural networks have been proposed to speed-up and automatise this procedure, obtaining promising results. With the advent of magnetic resonance imaging (MRI)-guided radiotherapy, MR-based segmentation is becoming increasingly relevant. However, the majority of the studies investigated automatic contouring based on computed tomography (CT).

**Purpose:**

In this study, we investigate the feasibility of clinical use of deep learning-based automatic OARs delineation on MRI.

**Materials and methods:**

We included 150 patients diagnosed with prostate cancer who underwent MR-only radiotherapy. A three-dimensional (3D) T1-weighted dual spoiled gradient-recalled echo sequence was acquired with 3T MRI for the generation of the synthetic-CT. The first 48 patients were included in a feasibility study training two 3D convolutional networks called DeepMedic and dense V-net (dV-net) to segment bladder, rectum and femurs. A research version of an atlas-based software was considered for comparison. Dice similarity coefficient, 95% Hausdorff distances (HD_95_), and mean distances were calculated against clinical delineations. For eight patients, an expert RTT scored the quality of the contouring for all the three methods. A choice among the three approaches was made, and the chosen approach was retrained on 97 patients and implemented for automatic use in the clinical workflow. For the successive 53 patients, Dice, HD_95_ and mean distances were calculated against the clinically used delineations.

**Results:**

DeepMedic, dV-net and the atlas-based software generated contours in 60 s, 4 s and 10-15 min, respectively. Performances were higher for both the networks compared to the atlas-based software. The qualitative analysis demonstrated that delineation from DeepMedic required fewer adaptations, followed by dV-net and the atlas-based software. DeepMedic was clinically implemented. After retraining DeepMedic and testing on the successive patients, the performances slightly improved.

**Conclusion:**

High conformality for OARs delineation was achieved with two in-house trained networks, obtaining a significant speed-up of the delineation procedure. Comparison of different approaches has been performed leading to the succesful adoption of one of the neural networks, DeepMedic, in the clinical workflow. DeepMedic maintained in a clinical setting the accuracy obtained in the feasibility study.

## Background

Structure delineation is a necessary, yet time-consuming manual procedure in radiotherapy. Consistent and accurate delineation of organs-at-risk (OARs) and target structures for prostate patients is vital when performing dose escalation and treating patients with highly conformal plans [1]. Traditionally, computed tomography (CT) has been used for radiotherapy simulation and structure delineation [2]. In the last few decades, magnetic resonance imaging (MRI) has found its way for radiotherapy simulation as it provides superior soft-tissue contrast compared to CT [3, 4], thus enabling more accurate delineation of target regions and critical structures compared to CT [5–7].

The manual segmentation of anatomical structures is a time-consuming process [8]. Besides, with the advent of MR-guided radiotherapy [9–11], the accuracy and speed of delineations become the weakest link [12] that hinders the possibilities of online adaptive radiotherapy by being responsible for longer fraction time [13].

To automatically perform delineations of target and OARs for patients affected by prostate cancer, various methods have been developed over the past years. For example, three-dimensional (3D) deformable model surface [14], organ-based modelling [15], and atlas-based solutions [16, 17] have been demonstrated. For all these methods, the time required to perform segmentation is in the order of minutes, if not hours, which is excessive to enable online adaptive treatments. To obviate this limitation, currently in online treatments only the target delineations and the OARs in the vicinity of the target (e.g. within a ring of 3-5 cm) are adjusted due to the excessive time needed for OARs segmentation [18–20].

Recently, deep learning has been proposed to speed-up and automatise automatic segmentation obtaining promising results [8, 21, 22]. Deep learning is a branch of artificial intelligence and machine learning that involves the use of neural networks to generate a hierarchical representation of the input data to achieve a specific task without the need of hand-engineered features [23, 24].

Many studies focused on target delineations [8] reaching mean dice similarity coefficients compared to manual delineations in the range 0.82-0.95 [25–31]. Automatic delineation of OARs is also a crucial aspect to achieve full online adaptive radiotherapy and to possibly save time to manual contouring.

In this study, we aim at investigating the feasibility of convolutional neural network-based automatic OARs delineation on MRI. A preliminary retrospective study was conducted to select a suitable network architecture and prepare for clinical implementation. After having chosen the most suitable convolutional network and performing clinical implementation, performances of automatic deep learning-based OARs delineation from our clinic are presented.

## Material and methods

### Patient data collection

Patients diagnosed with intermediate and high-risk prostate cancer undergoing MR-only radiotherapy [32] in the period between June 2018, and January 2020 were included in the study. Further inclusion criteria were: the presence of four gold fiducial markers for position verification and absence of hip implants. The patients were also scanned with a specific radio-frequency spoiled gradient-recalled echo (SPGR) sequence that will be described in more detail further on. The clinical exclusion criteria for MR-only radiotherapy were: patients with more than four positive lymph-nodes (N1, as on PET-CT or after pelvic lymph-nodes dissection), life expectancy <10 years (as from WHO >3), prior pelvic irradiation, IPSS >20, presence of prostatitis, active Crohn’s disease, colitis ulcerosa or diverticulitis, an anastomotic bowel in the high dose region and patients undergoing trans-rectal prostate resection less than three months before treatment. With the application of these exclusion criteria, a total of 150 patients that were included in this study and treated with external beam radiotherapy.

For all patients, 3T MRI (Ingenia MR-RT, v 5.3.1, Philips Healthcare, the Netherlands) was acquired after requesting the patients to empty their bladder and drink 200-300 ml of water one hour before the acquisition. Patients were positioned on a vendor-provided flat table using a knee support cushion (lower extremity positioning system, without adjustable FeetSupport, MacroMedics BV, the Netherlands). Patients were tattooed at the MRI with the aid of a laser system (Dorado3, LAP GmbH Laser Applikationen, Germany) to facilitate treatment positioning. Also, MR-visible markers (PinPoint *Ⓡ* for Image Registration 128, Beekley Medical, USA) were used to identify the set-up location on MRI. MR images were acquired using anterior and posterior phased array coils (dS Torso and Posterior coils, 28 channels, Philips Healthcare, the Netherlands). Two in-house-built bridges supported the anterior coil to avoid skin contour deformation.

**Fig. 1:**
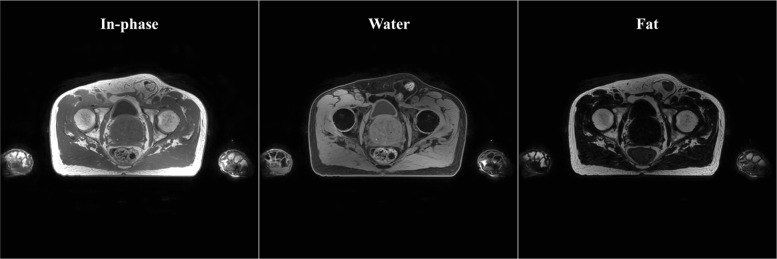
Transverse view of in-phase (IP), water (W) and fat (F) images for a patient (69 yo) diagnosed with T2b cancer. Note the large portion of void space surrounding the patient body. Cropping has been applied as preprocessing to remove such void regions

OARs were contoured on Dixon images [33] obtained with a dual-echo three-dimensional (3D) Cartesian radio-frequency SPGR sequence. For each patient, in-phase (IP), water (W), and fat (F) images [34] (Fig. 1) were reconstructed as in [35]. Dixon images were generated as part of a proprietary solution (MRCAT, rev. 257, Philips Healthcare, Finland) that enabled MR-based dose calculation for patients with prostate cancer [36, 37]. The imaging parameters, reported in Table 1, were locked by the vendor; therefore, they were stable through the whole study. Radiotherapy technicians (RTTs) with dedicated experience in contouring delineated bladder, rectum and femurs using IP, W and F Dixon images. The OARs delineations were approved or revised by a radiation oncologist. Besides, the radiation oncologist delineated the target structures. The delineation indications followed RTOG guidelines [38] requiring that the rectum was delineated from the outer part of the sphincter (anus) until the sigmoid fold (expected length of the rectum was 10-15 cm), as described in [39], with the sphincter delineated as a separate structure. The bladder was entirely delineated, while the femurs were delineated in the whole FOV of the image. In the case of regional radiotherapy, the bowel bag was also included.

**Table 1:** Image parameters of the sequences used for the OARs contouring. The term FOV refers to the field-of-view, while AP to anterior-posterior and LR to right-left

**Imaging parameters**	**Value**
**TE**_**1**_**/TE**_**2**_**/TR** [ms]	1.2/2.5/3.9
**Flip Angle** [ ^∘^]	10
**FOV**^∗^ [cm^3^]	55.2x55.2x30
**Acquisition Matrix**^∗^	324x324x120
**Reconstruction Matrix**^∗^	528x528x120
**Reconstructed Voxel**^∗^ [mm^3^]	1x1x2.5
**Bandwidth** [Hz/px]	1072
**Readout direction**	AP
**Phase direction**	RL
**Geometry correction**	3D
**Acquisition time**	2 min 17 s

^∗^expressed in terms of anterior-posterior (AP), right-left (RL) and superior-inferior directions

### Study design

The first 48 patients (treated until January 2019) were included in a feasibility study training two state-of-the-art 3D convolutional networks called DeepMedic [40] and dense V-net (dV-net) [41] (“Networks architecture, image processing and training” section). Three-fold cross-validation was performed, splitting the patients in 32/16 for train/validation. The network hyperparameters were optimised on the first fold and maintained for the other two folds. For example, the number of epochs was chosen considering the loss function in the validation set by performing early stopping when loss function did not decrease after five consecutive epochs.

The performance of the networks was compared against a research version of commercial software based on multi-atlases and deformable registration and against the clinically used delineations (“Evaluation” section).

**Fig. 2:**
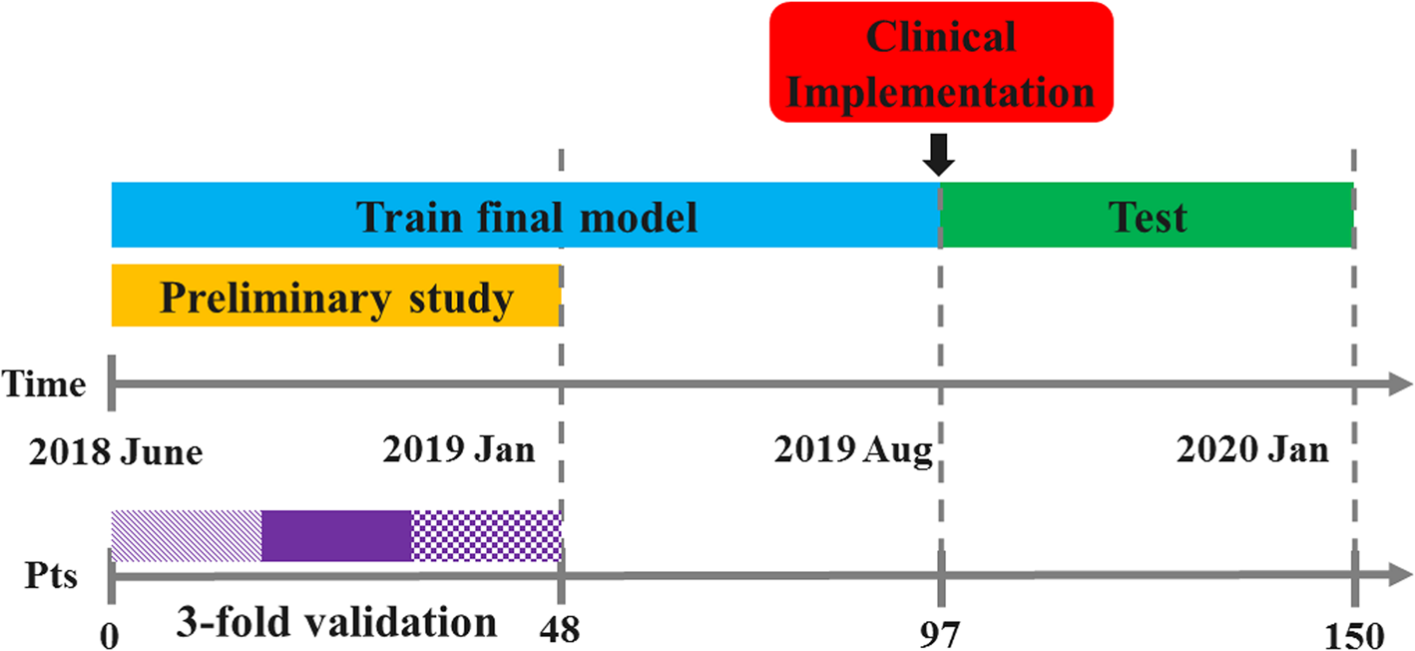
Schematic of the study design representing the timeline and the number of patients included. Also, the length and the number of patients for the preliminary study, the training of the final model and the patients used for testing the clinical implementation are reported

This preliminary study enabled us to choose among the three automatic methods. The preferred approach was retrained on 97 patients that were imaged and treated until August 2019; it was implemented for automatic use in the clinical workflow. The performances of the implemented model were reported on the 53 successive consecutively treated patients. A schematic overview of the study design is presented in Fig. 2.

### Networks architecture, image processing and training

Three-dimensional network architectures were chosen to investigate performance differences considering as perceptive field the whole volumes or smaller patches. In particular, DeepMedic [40] was the network chosen to perform patch-based training, while dV-net [41] was chosen to perform training on whole volumes. The two architectures, which will be described in detail in the next sections, required similar pre-processing. Three channels were used as input: IP, W and F images. The OARs that were considered as target are: bladder, rectum, right and left femur; they were decoded as masks with values from 1 to 4 without overlapping each others. To increase the amount of contextual information, the CTV was also decoded with a value of 5, which means that the networks also output CTV. Note that CTV was not considered in our study given that CTV delineation is clinically based on a different MRI, i.e. T2-weighted turbo spin-echo sequences [42]. The networks were trained on a graphical processing unit (GPU) Tesla P100 (NVIDIA Corporation, USA) with 16 GB of memory. To allow the whole volume to fit on the GPU, the IP, W and F images were initially cropped with 90 voxels at the borders of the anterior-posterior and lateral directions obtaining matrices of 348x348x120 voxels. Note that an observer controlled the presence of femurs within the FOV. Also, the image intensity of IP, W and F were clipped at their respective 99.9 percentile per each patient volume. Images were subsequently divided by the standard deviation (*σ*), and then a fixed value of 1 was subtracted.

After training and inference of the networks, the delineations were post-processed generating four binary volumes. Morphological operations of closure and hole filling by one voxel were applied. The largest 3D connected region was selected for each delineated structure. These operations were performed to remove possible small-sized delineations that may have been found by the networks.

#### DeepMedic

The DeepMedic [40] implementation employed was provided by the Kamnitsas et al.^1^ in Tensorflow v1.7. The model employed a three-pathway architecture for multi-resolution processing of 3D patches. A low, medium and high-resolution pathway with receptive fields of 85^3^, 51^3^, 17^3^ voxels were employed with each pathway consisting of 11-layers. A fully connected network (FCN) was used for combining the pathways and post-processing, as presented by Kamnitsas et al. [40]. Note that the size of the receptive fields has been modified compared to the original implementation.

The training configuration was kept as the original, with learning rate = 0.001, Adam optimiser with momentum = 0.6, epochs = 35, batch size = 10 and $\mathcal {L}_{1}$ and $\mathcal {L}_{2}$ regularisations 2 weighted with factor 0.000001 and 0.0001, respectively. The configuration file is reported in the Supplementary Material. All the OARs were equally sampled during training enforcing that the patches considered in each epoch contains the four OARs the same amount of times. Also, as in Kamnitsas et al. [40], volumetric dice similarity coefficient was adopted as the loss function. Data augmentation was applied in terms of random shifts and rescaling perturbation of the intensity (*I*) by the following: *I*^′^=(*I*+*s*)∗*m*, where *s* and *m* where Gaussian distributed with *μ*=0, 1 and *σ*=0.05, 0.01, respectively. For training, DeepMedic made use of about 9 GB of GPU memory.

#### Dense v-net

The dV-net implementation provided in NiftyNet was employed^3^. It consisted of a 3D U-Net with a sequence of three downsampling and dense upsampling feature strided stacks with skip connections to propagate higher resolution information to the final segmentation. Dilated convolutions were employed to reduce the number of features [41].

The training configuration was kept as the original, with learning rate= 0.001, Adam optimiser with momentum = 0.6, batch size = 6, $\mathcal {L}_{2}$ regularisation (weight = 0.001) and epoch = 25. The configuration file is reported in the Supplementary Material. Dice was adopted as loss function, and data augmentation was applied in terms of elastic deformation, as implemented within NiftyNet. For training, dV-net made use of about 16 GB of GPU memory.

### Evaluation

#### Preliminary study

The first 48 patients treated between June 2018 and August 2019 were included in a preliminary study to compare the performance of the two networks and atlas-based approach to the delineation used during clinical treatment planning.

The advanced medical imaging registration engine (ADMIRE, research version 1.13.5, Elekta AB, Sweden) was the software considered; ADMIRE is based on multi-atlases [43, 44] and gradient-free dense mutual information deformable registration [45]. In particular, the rectum was delineated based on the F image, bladder and femurs were delineated based on IP images using an atlas of 9 patients that were previously acquired with the same sequence. ADMIRE took about 10 to 15 minutes to generate automatic contouring on a Tesla K20c GPU (NVIDIA Corporation, USA) with 6 GB of memory.

Performances of the three automatic approaches were evaluated in terms of (volumetric) dice similarity coefficients (DSC), 95% boundary Hausdorff distances (HD_95_) [46], mean surface distance (MSD) against clinical delineations. All the metrics were calculated using Plastimatch^4^, except for the surface distance, which was calculated as from https://github.com/deepmind/surface-distance. In particular, violin plots [47] representing the mean, *σ*, 95% percentile and the probability distribution were obtained for the three metrics. Also, Wilcoxon signed-rank tests were conducted among the three evaluation metrics with a confidence interval of 0.05.

For a subset of 8 patients, an RTT with five years of experience in contouring scored the quality of the delineations for all three methods. The delineations were classified from zero to three, which corresponds to clinically acceptable, small modifications, large modifications, or clinically unacceptable contours. In total, the RTT scored 96 delineations. The percentage of each score over all the contours was reported for the three methods and visualised in a pie chart. Also, the most challenging structures (structures with an average score ≥2) were reported for each method.

#### Clinical implementation

After a choice was made among the three automatic approaches, the best performing network was retrained for the first 97 patients that were included up to August 2019. The hyperparameters were identical to the preliminary study. The network was implemented for clinical use complying with the medical device regulation (MDR 2017/745)^5^. Quantitative evaluation was perfomed in terms of DSC, HD_95_ and MSD for the 53 consecutive patients undergoing MR-only radiotherapy from August 2019 to January 2020. The delineations adopted for clinical use, i.e. delineated by RTTs and approved or re-adjusted by a radiation oncologist, were considered as reference. Also, surface dice similarity coefficient (SDSC) [48] was calculated^6^ to enable comparison with previous work [49]. Besides, the performance of the network clinically implemented was compared with the performance of the same network obtained during the preliminary study.

## Results

### Timing performance

The inference time of the network was about 60 s for DeepMedic and approximately 4 s for dV-net using the full resolution images of 328x328x120 voxels on GPU. ADMIRE generated contours in approximately 14 min on GPU.

### Preliminary study

**Fig. 3:**
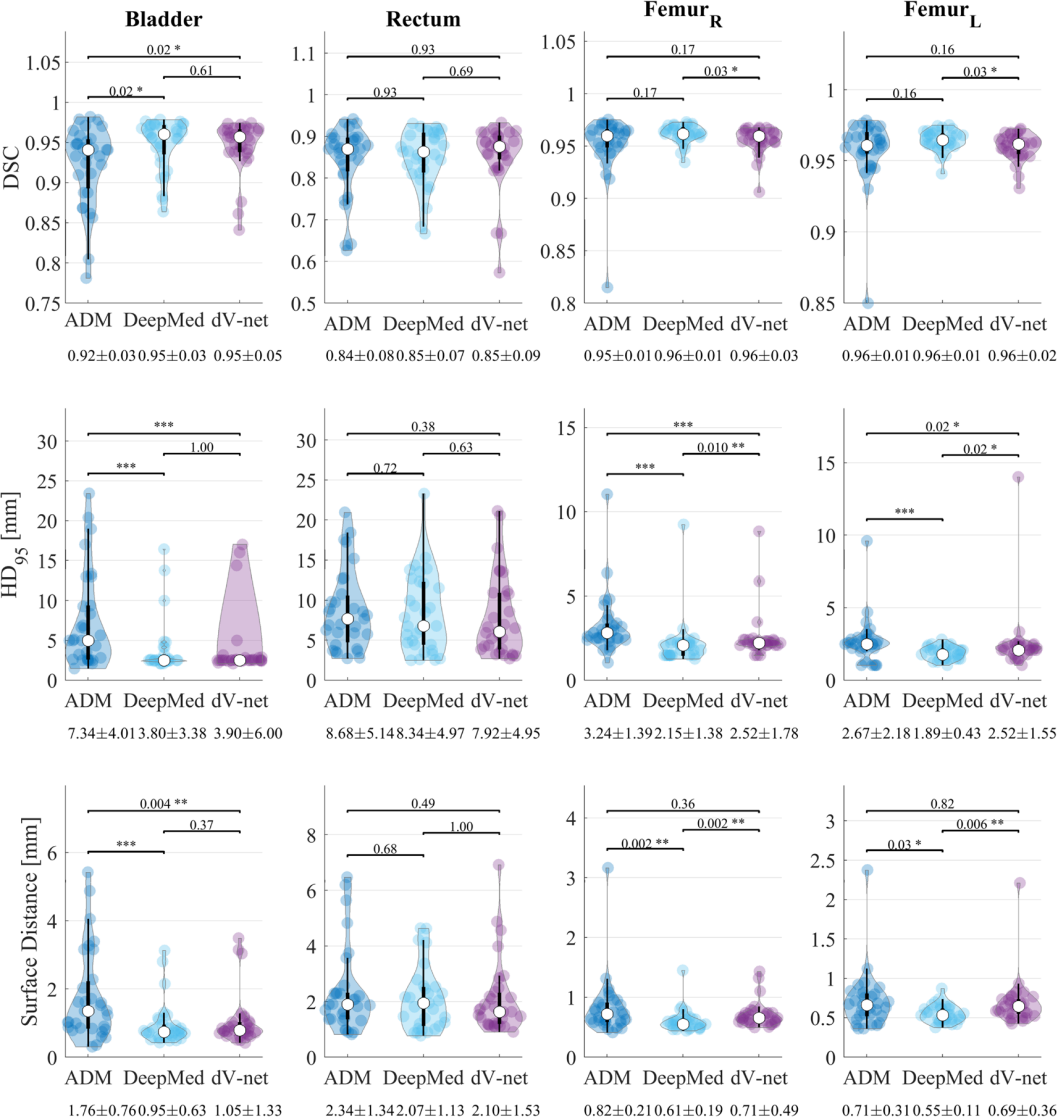
Violin plots representing the mean (white dot), *σ* (black vertical rectangle), 95% percentile (black vertical line) and the probability distribution for the dice similarity coefficient (DSC, top) and 95% Hausdorff distance (HD_95_, middle) and surface distance (bottom) for the OARs against clinical contours in among the preliminary study. The statistical significance of the Wilcoxon signed-rank test is reported as well as the mean(±*σ*) of each metric. The asterisks represent p ≤0.05 (∗), p ≤0.01 (∗∗) and p ≤0.001 (∗∗∗)

Figure 3 represents the violin plots for DSC, HD_95_ and the MSD. One can observe that performances were higher for both the networks compared to ADMIRE. For the bladder, no significant differences were observed between the networks, but significant differences were observed between the networks and ADMIRE. For the rectum, no significant differences were observed among the three automatic methods. When considering the femurs, DeepMedic outperformed both dV-net and ADMIRE. For example, for the right femur, the mean (±*σ*) HD_95_ was 2.2 ±1.4, 2.5 ±1.8 and 3.2 ±1.4 mm for DeepMedic, dV-net and ADMIRE, respectively.

**Fig. 4:**
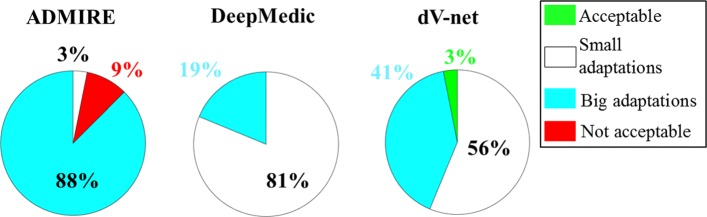
Pie chart reporting the percentage of the qualitative scoring performed by the expert RTT for each auto-segmentation method

The qualitative scoring by an RTT expert (Fig. 4) demonstrated that delineations from DeepMedic required fewer adaptations, followed by dV-net and then ADMIRE. Specifically, the expert RTT stated that, for all the structures, the number of delineations that were acceptable or needed small adjustment was 81%, 59% and 3% for DeepMedic, dV-net and ADMIRE, respectively. For both the networks, the rectum followed by bladder were indicated as the most challenging structures, while for ADMIRE, the bladder followed by rectum and femurs (same scoring) were the structures considered as the most challenging (score ≥ 2).

### Clinical implementation

On the basis of the preliminary analysis, we decided to implement DeepMedic for our clinic. Clinical implementation was performed in August 2019.

**Fig. 5:**
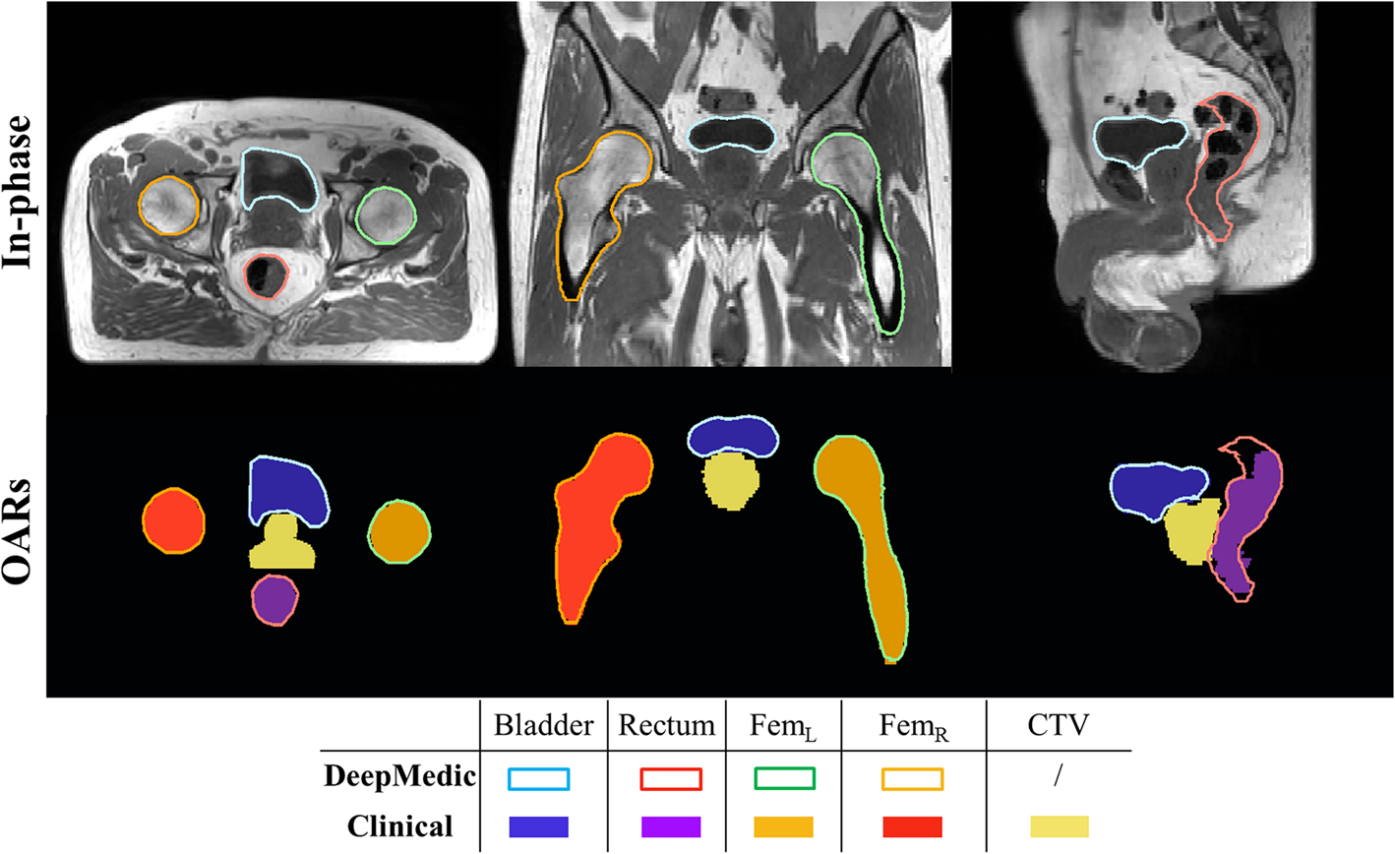
Example of in-phase MRI after cropping along with segmentations (OARs) obtained with DeepMedic (contours) versus clinical segmentations (filled contours) in the transverse (left), coronal (centre) and sagittal (right) view for a patient in the test. For this patient, average performance was obtained in terms of DSC: 0.96, 0.86, 0.97 and 0.97 for bladder, rectum, and femurs, respectively. Note that DeepMedic also outputs CTV, but it was not considered for clinical evaluation

The performance of DeepMedic in the preliminary study and after clinical implementation are presented in Table 2. After retraining DeepMedic and testing on the successive patients, the performances slightly improved. For example, it can be observed that, on average, the performance of DSC, HD_95_ and MSD after retraining the network on a more extensive set was ameliorated by 0.01-0.03, 1.2-1.4 mm and 0.1-0.4 mm, respectively. Delineations obtained with DeepMedic for a patient in the test set are presented in Fig. 5.

**Table 2:** Comparison of performance between the preliminary study (PS) and after the clinical implementation (Clinic) for DeepMedic in terms of (volumetric) dice similarity coefficient (DSC), 95% Hausdorff distance (HD_95_) and mean surface distance (MSD)

**Site**	DSC		HD_95_		MSD	
	PS	Clinic	PS	Clinic	PS	Clinic
			[mm]	[mm]	[mm]	[mm]
**Bladder**	0.95 ±0.03	0.96 ±0.02	3.8 ±3.4	2.5 ±1.1	1.0 ±0.6	0.6 ±0.3
**Rectum**	0.85 ±0.07	0.88 ±0.05	8.3 ±5.0	7.4 ±4.4	2.1 ±1.1	1.7 ±0.8
**Femur**_L_	0.96 ±0.01	0.97 ±0.01	2.2 ±1.4	1.6 ±0.5	0.6 ±0.2	0.5 ±0.1
**Femur**_R_	0.96 ±0.01	0.97 ±0.01	1.9 ±0.4	1.5 ±0.6	0.6 ±0.1	0.5 ±0.1

**Fig. 6:**
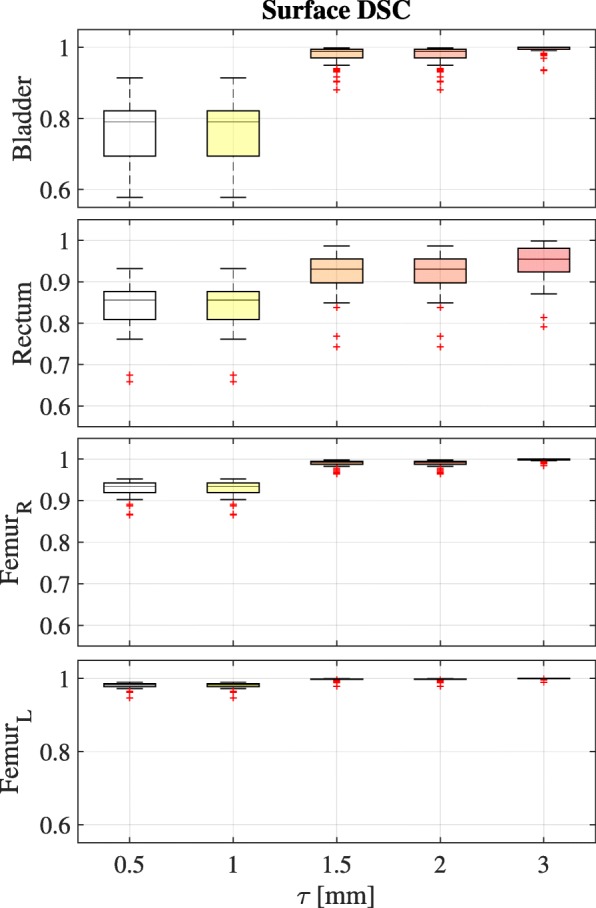
Boxplots for each structure of surface Dice similarity coefficient (SDSC) as a function of threshold (*τ*) for the 53 patients after clinial implementation. The data is plotted for the range of *τ* from sub-pixel (0.5 mm) to above the voxel size (3 mm). Box plots are shown with an inter-quartile range from 25 to 75% with the horizontal line representing mean value. Upper and lower whisker represent the 2.5 and 97.5 percentiles

Also, the SDSC was calculated for several threshold, *τ*= 0.5, 1, 1.5, 2 and 3 mm as reported in Fig. 6. The mean (±*σ*) DSCS was 0.98 ±0.03, 0.92 ±0.05, 0.989 ±0.008 and 0.997 ±0.003 for *τ*=2 mm for bladder, rectum, left and right femur, respectively.

## Discussion

The use of MRI for prostate radiotherapy delineation is becoming increasingly common among radiotherapy departments [50]. MRI are used to plan radiotherapy [32, 51]. Besides, use of MRI is also accelerated by the adoption of new advancements in linear accelerator technology, whereby daily MR imaging in treatment position is possible [9–11].

In this study, we demonstrated that deep learning-based approaches can utilise MRI to automatically segment OARs achieving high conformality. Also, a convolutional network has been implemented for clinical use, demonstrating the capability to maintain the performances obtained in a preliminary study.

Table 3 compiles previous work based on the use of convolutional networks and a selection of conventional approaches [16, 17, 52] for OARs delineation in the pelvic area. One can notice that CT-based segmentation [53–55] achieved mean DSC in the range 0.88-0.95 for prostate, rectum and bladder. Also, MRI-based segmentation [27, 49, 56] achieved mean DSC in the range 0.82-0.95. This study seems to outperform previous studies in almost all the metrics (in bold in the Table) except for the rectum, as obtained by Kazemifar et al. [54] and the HD_95_ and MSD as obtained by Kazemifar et al. and Dong et al. [56]. Comparing the results of automated contouring methods should be done with caution. For example, the guidelines used for clinical delineation may be different, and the impact of inter-observer variability on deep learning-based methods is not generally investigated [57]. In this sense, our study is novel given that a comparison of approaches based on CNNs to an atlas-based method is presented.

**Table 3:** Overview of the performance of automatic OARs delineations based on MRI and CT subdivided in convolutional network-based and conventional approaches. The number of patients included in the study (Pts), the imaging modality, a brief description of the method and metrics as dice similarity coefficient (DSC), 95% boundary Hausdorff distance (HD_95_) and mean surface distance (MSD) were reported for each study. HD_95_ and MSD are expressed in mm

**Study**	**Pts**	**Modality**	**Method(s)**	**Bladder**	**Rectum**	**Femur**_**L**_	**Femur**_**R**_
				DSC	DSC	DSC	DSC
				HD_95_	HD_95_	HD_95_	HD_95_
				MSD	MSD	MSD	MSD
**Convolutional network-based**							
Men2017 [53]	218/60 ^∗^	CT	2D	0.92		0.93	0.92
			dilated				
			VGG-16				
Feng2018 [27]	30/10 ^∗^	MRI	Multi-task	0.952 ±0.007	0.88 ±0.03		
			residual				
			2D FCN				
Kazemifar2018 [54]	51/9/20 ^∗^	CT	2D	0.95 ±0.04	**0.92±0.06**		
			U-net	**0.4±0.6**	**0.2±0.3**		
				1.1 ±0.8^*a*^	**0.8±0.**6^*a*^		
Balagopal2018 [55]	108/28	CT	2D U-net	0.95 ±0.02	0.84 ±0.04	0.96 ±0.03	0.95 ±0.01
	mean		+ 3D U-net	17.0 ±14.6	4.9 ±3.9		
	4 models		(ResNeXT)	0.5 ±0.7	0.8 ±0.7		
Dong2019 [56]	140x5 ^+^	MRI	3D Cycle-GAN	0.95 ±0.03	0.89 ±0.04		
			+ deep attention	6.81 ±9.25	10.84 ±15.59		
			U-net	**0.52±0.22**	0.92 ±1.03		
Elguindi2019 [49]	40/10/50	MRI		0.93 ±0.04	0.82 ±0.05		
			DeepLabV3+				
				0.92 ±0.1^*b*^	0.87 ±0.07^*b*^		
**This study**	97/53 ^∗^	MRI	3D	**0.96±0.02**	0.88 ±0.05	**0.97±0.01**	**0.97±0.01**
			multi-scale	2.5 ±1.1	7.4 ±4.4	**1.6±0.5**	**1.5±0.5**
			DeepMedic	**0.6±0.3**	1.7 ±0.8	**0.5±0.1**	**0.5±0.1**
				0.98 ±0.03^*c*^	0.92 ±0.05^*c*^	**0.989±0.008**^*c*^	**0.997±0.003**^*c*^
**Conventional**							
LaMacchia2012 [16]	5	CT	ABAS 2.0	0.93 ±0.03	0.77 ±0.07	0.94 ±0.04	0.94 ±0.04
			VelocityAI 2.6.2	0.72 ±0.15	0.75 ±0.04	0.92 ±0.02	0.92 ±0.03
			MIM 5.1.1	0.93 ±0.02	0.87 ±0.05	0.94 ±0.02	0.94 ±0.01
Dowling2015 [17]	39	MRI	multi-atlas	0.86 ±0.12	0.84 ±0.06	0.91 ±0.03	
			voting				
			diffeomorphic reg	5.1 ±4.6	2.4 ±1.0	1.5 ±0.5	
Delpon2016 [52]	10/10 ^∗^	CT	Mirada	0.76 ±0.12	0.73 ±0.07	0.89 ±0.05	0.91 ±0.03
				15 ±9	10 ±3	0.2 ±6.4	8.1 ±5.6
			MIM	0.80 ±0.14	0.75 ±0.07	0.89 ±0.08	0.92 ±0.02
				14.0 ±6.3	9.9 ±3.4	9.9 ±7.9	8.2 ±5.3
			ABAS	0.81 ±0.13	0.75 ±0.09	0.91 ±0.04	0.92 ±0.02
				13.6 ±7.9	9.9 ±4.4	8.6 ±6.9	8.5 ±6.1
			SPICE	0.76 ±0.26	0.68 ±0.12	0.70 ±0.05	0.72 ±0.03
				9.2 ±11.7	13 ±5	29.7 ±9.0	30 ±6.5
			Raystation	0.59 ±0.15	0.49 ±0.12	0.91 ±0.03	0.92 ±0.02
				28.5 ±13.1	16.5 ±3.7	8.8 ±7.2	6.4 ±5.0

^∗^ training/(validation)/test; ^+^ indicating x... cross-fold validation; ^*a*^ mean surface Hausdorff distance; ^*b*,*c*^ surface dice similarity coefficient as in [48] with *τ*=3 or 2 mm, respectively

In this study, a qualitative assessment by a manual observer has been presented. Unfortunately, it has not been recorded whether the overall time for the delineation has been reduced. Previous studies investigated this aspect [58] when introducing deep learning-based techniques in their clinic. Also, it is unclear whether the performance of the network may further improve when a dataset larger than 97 patients is used for training. This may be an object of future research.

The time necessary for automatic delineation on full FOV is within a minute. Such time-scale can be of interest for conventional radiotherapy and for MR-guided treatments. On the one hand, for conventional radiotherapy, fast automatic OAR segmentation may facilitate the reducing delays in the start of the treatments that may lead to hampered clinical outcomes [59]. On the other hand, for online adaptive MR-guided radiotherapy, fast OAR segmentation may relieve clinicians from dedicating effort in OARs segmentation while facilitating the delineation of the target [60]. Currently, it has been reported that about 5-10 min is necessary for the for delineation in an online setting [19]. The time frame reported in our work may facilitate online adaptive radiotherapy, especially with an integrated automatic workflow.

## Conclusion

High conformality for OARs delineation was achieved with two in-house trained networks, obtaining a significant speed-up of the delineation procedure. One of the networks, DeepMedic, was successfully adopted in the clinical workflow maintaining in the clinical setting the accuracy obtained in the feasibility study conducted before clinical implementation.

## Supplementary information

**Additional file 1** Configuration files for the network architectures. As part of the supplementary material, it is possible to download the configuration files (ConfigFiles.zip) for DeepMedic (DeepMedic_model.cfg, DeepMedic_inference.cfg, DeepMedic_train.cfg) and dV-net (NiftyNet_train.ini). The configuration files for DeepMedic are three and contains the information regarding the model (DeepMedic_model.cfg), training (DeepMedic_train.cfg) and inference (DeepMedic_inference.cfg) of the network.

## Data Availability

The datasets analysed during the current study are not publicly available due to the internal policy of the Medical Ethical Commission about data sharing. The configuration files of DeepMedic and dV-net are reported as Supplementary Materials.

## Abbreviations

OAR(s): organ(s)-at-risk
MR(I): magnetic resonance (imaging)
CTV: clinical target volume
3D: three-dimensional
SPGR: spoiled gradient-recalled echo
PET: positron emission tomography
WHO: world health organisation
IPSS: international prostate symptom score
IP: in-phase
W: water
F: fat
FOV: field-of-view
TE: echo time
TR: repetition time
AP: anterior-posterior
RL: right-left
RTT: radiotherapy technician
dV-net: dense V-net
GPU: graphical processing unit
FCN(s): fully connected networks
ADMIRE: advanced medical imaging registration engine
DSC: dice similarity coefficient
MSD: mean surface distance
HD_95_: 95% boundary Hausdorff distance
SDSC: surface dice similarity coefficient

## Footnotes

1 as available at https://github.com/Kamnitsask/deepmedic.

2 Consult the https://niftynet.readthedocs.io/en/dev/config_spec.html#reg-type for understanding the regularisation.

3 as available at https://github.com/NifTK/NiftyNet.

4 as available at https://plastimatch.org/

5 European regulation 2017/745 of the European Parliament and of the Council of 5 April 2017 on medical devices, https://eur-lex.europa.eu/eli/reg/2017/745/oj.

6 as available in https://github.com/deepmind/surface-distance.

## References

[1] Salembier C, Villeirs G, De Bari B, Hoskin P, Pieters BR, Van Vulpen M, Khoo V, Henry A, Bossi A, De Meerleer G, Fonteyne V (2018). ESTRO ACROP consensus guideline on CT- and MRI-based target volume delineation for primary radiation therapy of localized prostate cancer. Radiother Oncol.

[2] Boehmer D, Maingon P, Poortmans P, Baron MH, Miralbell R, Remouchamps V, Scrase C, Bossi A, Bolla M (2006). Guidelines for primary radiotherapy of patients with prostate cancer. Radiother Oncol.

[3] Debois M, Oyen R, Maes F, Verswijvel G, Gatti G, Bosmans H, Feron M, Bellon E, Kutcher G, Van Poppel H, Vanuytsel L (1999). The contribution of magnetic resonance imaging to the three-dimensional treatment planning of localized prostate cancer,. Int J Radiat Oncol Biol Phys.

[4] Dirix P, Haustermans K, Vandecaveye V (2014). The Value of Magnetic Resonance Imaging for Radiotherapy Planning,. Semin Radiat Oncol.

[5] Roach MI, Faillace-Akazawa P, Malfatti C, Holland J, Hricak H (1996). Prostate volumes defined by magnetic resonance imaging and computerized tomographic scans for three-dimensional conformal radiotherapy. Int J Radiat Oncol Biol Phys.

[6] Rasch C, Barillot I, Remeijer P, Touw A, van Herk M, Lebesque JV (1999). Definition of the prostate in CT and MRI: a multi-observer study,. Int J Radiat Oncol Biol Phys.

[7] Villeirs GM, Vaerenbergh K, Vakaet L, Bral S, Claus F, Neve WJ, Verstraete KL, Meerleer GO (2005). Interobserver Delineation Variation Using CT versus Combined CT + MRI in Intensity-Modulated Radiotherapy for Prostate Cancer. Strahlenther Onkol.

[8] Cardenas CE, Yang J, Anderson BM, Court LE, Brock KB (2019). Advances in Auto-Segmentation. Sem Radiat Oncol.

[9] Raaymakers BW, Raaijmakers AJE, Kotte ANTJ, Jette D, Lagendijk JJW (2004). Integrating a MRI scanner with a 6 MV radiotherapy accelerator: dose deposition in a transverse magnetic field. Phys Med Biol.

[10] Dempsey J, Benoit D, Fitzsimmons J, Haghighat A, Li J, Low D, Mutic S, Palta J, Romeijn H, Sjoden G (2005). A device for realtime 3D image-guided IMRT,. Int J Radiat Oncol Biol Phys.

[11] Fallone BG, Murray B, Rathee S, Stanescu T, Steciw S, Vidakovic S, Blosser E, Tymofichuk D (2009). First MR images obtained during megavoltage photon irradiation from a prototype integrated linac-MR system,. Med Phys.

[12] Njeh CF (2008). Tumor delineation: The weakest link in the search for accuracy in radiotherapy. J Med Phys.

[13] Keall P, Poulsen P, Booth JT (2019). See, Think, and Act: Real-Time Adaptive Radiotherapy. Sem Radiat Oncol.

[14] Pekar V, McNutt TR, Kaus MR (2004). Automated model-based organ delineation for radiotherapy planning in prostatic region. Int J Radiat Oncol Biol Phys.

[15] Pasquier D, Lacornerie T, Vermandel M, Rousseau J, Lartigau E, Betrouni N (2007). Automatic Segmentation of Pelvic Structures From Magnetic Resonance Images for Prostate Cancer Radiotherapy. Int J Radiat Oncol Biol Phys.

[16] La Macchia M, Fellin F, Amichetti M, Cianchetti M, Gianolini S, Paola V, Lomax AJ, Widesott L (2012). Systematic evaluation of three different commercial software solutions for automatic segmentation for adaptive therapy in head-and-neck, prostate and pleural cancer. Radiat Oncol.

[17] Dowling JA, Sun J, Pichler P, Rivest-Hénault D, Ghose S, Richardson H, Wratten C, Martin J, Arm J, Best L, Chandra SS, Fripp J, Menk FW, Greer PB (2015). Automatic Substitute Computed Tomography Generation and Contouring for Magnetic Resonance Imaging (MRI)-Alone External Beam Radiation Therapy From Standard MRI Sequences. Int J Radiat Oncol Biol Phys.

[18] Raaymakers BW, Jürgenliemk-Schulz IM, Bol GH, Glitzner M, Kotte ANTJ, van Asselen B, de Boer JCJ, Bluemink JJ, Hackett SL, Moerland MA, Woodings SJ, Wolthaus JWH, van Zijp HM, Philippens MEP, Tijssen R, Kok JGM, de Groot-van Breugel EN, Kiekebosch I, Meijers LTC, Nomden CN, Sikkes GG, Doornaert PAH, Eppinga WSC, Kasperts N, Kerkmeijer LGW, Tersteeg JHA, Brown KJ, Pais B, Woodhead P, Lagendijk JJW (2017). First patients treated with a 1.5 T MRI-Linac: clinical proof of concept of a high-precision, high-field MRI guided radiotherapy treatment. Phys Med Biol.

[19] Werensteijn-Honingh AM, Kroon PS, Winkel D, Aalbers EM, van Asselen B, Bol GH, Brown KJ, Eppinga WSC, van Es CA, Glitzner M, de Groot-van Breugel EN, Hackett SL, Intven M, Kok JGM, Kontaxis C, Kotte AN, Lagendijk JJW, Philippens MEP, Tijssen RHN, Wolthaus JWH, Woodings SJ, Raaymakers BW, Jurgenliemk-Schulz IM (2019). Feasibility of stereotatctic radiotherapy using a 1.5T MR-linac: Multi-fraction treatment of pelvic lymph node oligometastases. Radiother Oncol.

[20] Bruynzeel AM, Tetar SU, Oei SS, Senan S, Haasbeek CJ, Spoelstra FO, Piet AH, Meijnen P, van der Jagt MAB, Fraikin T (2019). A prospective single-arm phase 2 study of stereotactic magnetic resonance guided adaptive radiation therapy for prostate cancer: early toxicity results. Int J Radiat Oncol Biol Phys.

[21] Ronneberger O, Fischer P, Brox T. U-net: Convolutional networks for biomedical image segmentation. In: International Conference on Medical Image Computing and Computer-assisted Intervention. Springer: 2015. p. 234–41. 10.1007/978-3-319-24574-4_28.

[22] Milletari F, Navab N, Ahmadi S-A. V-net: Fully convolutional neural networks for volumetric medical image segmentation. In: 2016 Fourth International Conference on 3D Vision (3DV). IEEE: 2016. p. 565–71. 10.1109/3dv.2016.79.

[23] Meyer P, Noblet V, Mazzara C, Lallement A (2018). Survey on deep learning for radiotherapy. Comp Biol Med.

[24] Sahiner B, Pezeshk A, Hadjiiski LM, Wang X, Drukker K, Cha KH, Summers RM, Giger ML (2019). Deep learning in medical imaging and radiation therapy. Med Phys.

[25] Alvarez C, Martínez F, Romero E (2017). A multiresolution prostate representation for automatic segmentation in magnetic resonance images. Med Phys.

[26] Fu Y, Mazur TR, Wu X, Liu S, Chang X, Lu Y, Li HH, Kim H, Roach MC, Henke L, Yang D (2018). A novel MRI segmentation method using CNN-based correction network for MRI-guided adaptive radiotherapy. Med Phys.

[27] Feng Z, Nie D, Wang L, Shen D. Semi-supervised learning for pelvic MR image segmentation based on multi-task residual fully convolutional networks. IEEE Comput Soc. 2018. 10.1109/ISBI.2018.8363713. 10.1109/ISBI.2018.8363713 PMC6193482 30344892

[28] Nie D, Wang L, Gao Y, Lian J, Shen D. STRAINet: Spatially Varying sTochastic Residual AdversarIal Networks for MRI Pelvic Organ Segmentation. Trans Neural Netw Learn Syst IEEE. 2018:1–13. 10.1109/TNNLS.2018.2870182. 10.1109/TNNLS.2018.2870182 PMC6550324 30307879

[29] Liu C, Gardner SJ, Wen N, Elshaikh MA, Siddiqui F, Movsas B, Chetty IJ (2019). Automatic segmentation of the prostate on CT images using deep neural networks (DNN). Int J Radiat Oncol Biol Phys.

[30] Eppenhof KAJ, Maspero M, Savenije MHF, de Boer JCJ, van der Voort van Zyp JRN, Raaymakers BW, Raaijmakers AJE, Veta M, van den Berg CAT, Pluim JPW (2019). Fast contour propagation for MR-guided prostate radiotherapy using convolutional neural networks. Med Phys.

[31] Wang B, Lei Y, Tian S, Wang T, Liu Y, Patel P, Jani AB, Mao H, Curran WJ, Liu T, Yang X (2019). Deeply supervised 3D fully convolutional networks with group dilated convolution for automatic MRI prostate segmentation. Med Phys.

[32] Kerkmeijer LGW, Maspero M, Meijer GJ, van der Voort van Zyp JRN, de Boer HCJ, van den Berg CAT (2018). Magnetic Resonance Imaging only Workflow for Radiotherapy Simulation and Planning in Prostate Cancer. Clin Oncol.

[33] Dixon WT (1984). Simple proton spectroscopic imaging. Radiology.

[34] Eggers H, Brendel B, Duijndam A, Herigault G (2011). Dual-echo Dixon imaging with flexible choice of echo times. Magn Reson Med.

[35] Maspero M, Savenije MH, Dinkla AM, Seevinck PR, Intven MP, Jurgenliemk-Schulz IM, Kerkmeijer LG, van den Berg CA (2018). Dose evaluation of fast synthetic-ct generation using a generative adversarial network for general pelvis mr-only radiotherapy. Phys Med Biol.

[36] Maspero M, Seevinck PR, Schubert G, Hoesl MAU, van Asselen B, Viergever MA, Lagendijk JJW, Meijer GJ, van den Berg CAT (2017). Quantification of confounding factors in MRI-based dose calculations as applied to prostate IMRT. Phys Med Biol.

[37] Maspero M, Tyyger MD, Tijssen RH, Seevinck PR, Intven MP, van den Berg CA (2018). Feasibility of magnetic resonance imaging-only rectum radiotherapy with a commercial synthetic computed tomography generation solution. Phys Imag Radiat Oncol.

[38] Gay HA, Barthold HJ, O’Meara E, Bosch WR, El Naqa I, Willett C, Kachnic LA, Jhingran A, Portelance L, Ryu J, Small W, Gaffney D, Viswanathan AN, Michalski JM (2012). Pelvic Normal Tissue Contouring Guidelines for Radiation Therapy: A Radiation Therapy Oncology Group Consensus Panel Atlas. Int J Radiat Oncol Biol Phys.

[39] D’Souza N, de Neree tot Babberich MPM, Lord A, Shaw A, Abulafi M, Tekkis P, Wiggers T, Brown G (2018). The rectosigmoid problem. Surg Oncol.

[40] Kamnitsas K, Ferrante E, Parisot S, Ledig C, Nori AV, Criminisi A, Rueckert D, Glocker B. DeepMedic for Brain Tumor Segmentation. In: Brainlesion: Glioma, Multiple Sclerosis, Stroke and Traumatic Brain Injuries: Second International Workshop, BrainLes 2016, with the Challenges on BRATS, ISLES and mTOP 2016, Held in Conjunction with MICCAI 2016, Athens, Greece, October 17, 2016, Revised Selected Papers, vol. 10154. Springer: 2017. p. 138. 10.1007/978-3-319-55524-9.

[41] Gibson E, Giganti F, Hu Y, Bonmati E, Bandula S, Gurusamy K, Davidson B, Pereira SP, Clarkson MJ, Barratt DC. Automatic multi-organ segmentation on abdominal CT with dense v-networks. IEEE Trans Med Imaging. 2018; 37(8):1822–34. https://doi.org/10.1109%2FTMI.2018.2806309. 10.1109/TMI.2018.2806309 PMC6076994 29994628

[42] Coakley FV, Oto A, Alexander LF, Allen BC, Davis BJ, Froemming AT, Fulgham PF, Hosseinzadeh K, Porter C, Sahni VA, Schuster DM, Showalter TN, Venkatesan AM, Verma S, Wang CL, Remer EM, Eberhardt SC (2017). ACR Appropriateness Criteria® Prostate Cancer—Pretreatment Detection, Surveillance, and Staging. J Am Col Radiol.

[43] Han X. Learning-boosted label fusion for multi-atlas auto-segmentation. In: Lecture Notes in Computer Science (including Subseries Lecture Notes in Artificial Intelligence and Lecture Notes in Bioinformatics), vol. 8184 LNCS. Springer: 2013. p. 17–24. 10.1007/978-3-319-02267-3.

[44] Van de Velde J, Wouters J, Vercauteren T, De Gersem W, Achten E, De Neve W, Van Hoof T. Optimal number of atlases and label fusion for automatic multi-atlas-based brachial plexus contouring in radiotherapy treatment planning. Radiat Oncol. 2016; 11(1). 10.1186/s13014-015-0579-1. 10.1186/s13014-015-0579-1 PMC4705618 26743131

[45] Han X, Hibbard LS, Willcut V. GPU-accelerated, gradient-free MI deformable registration for atlas-based MR brain image segmentation. In: 2009 IEEE Computer Society Conference on Computer Vision and Pattern Recognition Workshops: 2009. p. 141–8. 10.1109/CVPRW.2009.5204043.

[46] Beauchemin M, Thomson KPB, Edwards G (1998). On the hausdorff distance used for the evaluation of segmentation results. Canad J Remote Sens.

[47] Hintze JL, Nelson RD (1998). Violin plots: a box plot-density trace synergism. Am Stat.

[48] Nikolov S, Blackwell S, Mendes R, De Fauw J, Meyer C, Hughes C, Askham H, Romera-Paredes B, Karthikesalingam A, Chu C, Carnell D, Boon C, D’Souza D, Moinuddin SA, Sullivan K, Consortium DR, Montgomery H, Rees G, Sharma R, Suleyman M, Back T, Ledsam JR, Ronneberger O. Deep learning to achieve clinically applicable segmentation of head and neck anatomy for radiotherapy. 2018. http://arxiv.org/abs/1809.04430.

[49] Elguindi S, Zelefsky MJ, Jiang J, Veeraraghavan H, Deasy JO, Hunt MA, Tyagi N (2019). Deep learning-based auto-segmentation of targets and organs-at-risk for magnetic resonance imaging only planning of prostate radiotherapy. Phys Imag Radiat Oncol.

[50] Schmidt MA, Payne GS (2015). Radiotherapy planning using MRI,. Phys Med Biol.

[51] Edmund JM, Nyholm T (2017). A review of substitute CT generation for MRI-only radiation therapy. Radiat Oncol.

[52] Delpon G, Escande A, Ruef T, Darréon J, Fontaine J, Noblet C, Supiot S, Lacornerie T, Pasquier D. Comparison of Automated Atlas-Based Segmentation Software for Postoperative Prostate Cancer Radiotherapy. Front Oncol. 2016; 6. 10.3389/fonc.2016.00178. 10.3389/fonc.2016.00178 PMC4971890 27536556

[53] Men K, Dai J, Li Y (2017). Automatic segmentation of the clinical target volume and organs at risk in the planning CT for rectal cancer using deep dilated convolutional neural networks. Med Phys.

[54] Kazemifar S, Balagopal A, Nguyen D, McGuire S, Hannan R, Jiang S, Owrangi A (2018). Segmentation of the prostate and organs at risk in male pelvic CT images using deep learning. Biomed Phys Eng.

[55] Balagopal A, Kazemifar S, Nguyen D, Lin M-H, Hannan R, Owrangi A, Jiang S (2018). Fully automated organ segmentation in male pelvic CT images. Phys Med Biol.

[56] Dong X, Lei Y, Tian S, Wang T, Patel P, Curran WJ, Jani AB, Liu T, Yang X. Synthetic MRI-aided multi-organ segmentation on male pelvic CT using cycle consistent deep attention network. Radiother Oncol. 2019. 10.1016/j.radonc.2019.09.028. 10.1016/j.radonc.2019.09.028 PMC6899191 31630868

[57] Sharp G, Fritscher KD, Pekar V, Peroni M, Shusharina N, Veeraraghavan H, Yang J. Vision 20/20: Perspectives on automated image segmentation for radiotherapy. Med Phys. 2014; 41(5). 10.1118/1.4871620@10.1002. 10.1118/1.4871620 PMC4000389 24784366

[58] Lustberg T, van Soest J, Gooding M, Peressutti D, Aljabar P, van der Stoep J, van Elmpt W, Dekker A (2018). Clinical evaluation of atlas and deep learning based automatic contouring for lung cancer. Radiother Oncol.

[59] Huang J, Barbera L, Brouwers M, Browman G, Mackillop WJ (2003). Does delay in starting treatment affect the outcomes of radiotherapy? A systematic review,. J Clin Oncol Off J Am Soc Clin Oncol.

[60] Keall PJ, Barton M, Crozier S (2014). The Australian magnetic resonance imaging-linac program. Semin Radiat Oncol.

